# From Flower to Honey Bouquet: Possible Markers for the Botanical Origin of *Robinia* Honey

**DOI:** 10.1155/2014/547275

**Published:** 2014-11-12

**Authors:** Giovanna Aronne, Manuela Giovanetti, Raffaele Sacchi, Veronica De Micco

**Affiliations:** ^1^Department of Agricultural and Food Sciences, University of Naples Federico II, Via Università 100, 80055 Portici, Italy; ^2^Department of Biology, University of Florence, Florence, Italy

## Abstract

Flowers are complex structures devoted to pollinator attraction, through visual as well as chemical signals. As bees collect nectar on flowers to produce honey, some aspects of floral chemistry are transferred to honey, making chemical markers an important technique to identify the botanical and geographical origins of honey. We applied a new approach that considers the simultaneous analysis of different floral parts (petals, stamens + pistils, calyxes + nectarines, and nectar) and the corresponding unifloral honey. We collected fresh flowers of *Robinia pseudoacacia* L. (black locust), selected five samples of *Robinia* honey from different geographical origins, applied SPME-GC/MS for volatile analyses, and defined the chemical contribution added by different floral parts to the honey final bouquet. Our results show that honey blends products from nectar as well as other flower parts. Comparing honey and flower profiles, we detected compounds coming directly from flower parts but not present in the nectar, such as hotrienol and *β*-pinene. These may turn out to be of special interest when selecting floral markers for the botanical origin of honey.

## 1. Introduction

One of the ways that globalization is affecting food markets is by increasing concern for the authenticity of original products [1]. Honey is a flavor-rich product, created by bees through an active search and collection of nectar from flowers. Depending on the visited flowers and on the climatic conditions at which nectar was produced, honey may taste quite differently. Over the years, customers have developed specific preferences for honeys of precise botanical and geographical origins and their demand supports local agricultural economies. Unfortunately, up until now, the accepted procedures to verify the botanical and geographical origins of honey have been based on complicate and expensive methods. The most traditional is melissopalynology, which consists of microscopic examination of pollen grains contained in a sample. It is the most reliable method, but it is also time consuming and requires a highly trained analyst. Efforts to limit the subjectivity of the analyst have been done by integrating traditional melissopalynology with multivariate statistical analysis [2]. Melissopalynology is often integrated by other methods, such as the determination of physicochemical parameters (color, free acidity, sugar contents, diastase activity, electrical conductivity, and specific rotation [3]) and sensory analysis [4], another procedure which requires specialized personnel. Therefore, the present technological state of honey analysis has the potential to resolve issues of product origin, but not to counterbalance the needs generated by globalization: informing policy and providing guidelines for applying standards to the market.

Aroma profiles are among the most typical features of food products. Volatile compounds are the main factors responsible for aroma. The identification of volatile compounds as possible markers of honeys has raised interest recently, and many works have since started investigating this topic (reviewed in [1, 5, 6]). There is still no unanimous agreement on how to select among the recognized compounds and until now, authors adopted different methods to extract the volatile fraction of honey [1] and different approaches to consider the relative importance of the resulting compounds. Radovic et al. [7] focused on similarities and differences among a range of honey samples to detect those elements characteristics of single honey types or origins, applying GC-MS (gas chromatography-mass spectrometry) after volatile extraction. Truchado et al. [8] analyzed the phenolics of a single unifloral honey and of the nectar collected from flowers of its hypothesized floral source, by means of SPE (solid phase extraction). Cuevas-Glory et al. [6] underlined the importance of avoiding, if possible, methods involving the use of solvents and/or sample heating or the isolation of volatiles from other major honey components, such as sugars and water, which could influence the final results. Some authors [6, 9] suggested that the best method for honey analyses is SPME-GC/MS (solid phase microextraction, followed by gas chromatography coupled to mass spectrometry). Lately, this technique has been successfully applied to honey volatiles identification. It is a simple, affordable, and effective tool that if improved may be of great help to differentiate honeys [6].

Flowers are complex structures. An Angiosperm hermaphrodite flower is composed of petals, pistil, stamens with pollen, and nectaries with nectar. All these parts may play an important role in attracting foraging bees and may be the source of volatile compounds that ultimately end up in honey [10]. Few attempts at coupling flower fragrance [11] or nectar aroma [8] to honey volatiles exist in the literature, and in these works only some flower parts have been taken into account, independently.

The aim of our study was to investigate the relative importance of contrasting flower structures involved in honey bee attraction and possibly contributing to corresponding honey aromas. We used* Robinia pseudoacacia* L. as a case study. Honey bees are attracted by flowers of* R. pseudoacacia* [12] and produce a honey known world-wide for its delicate fragrance. We compared the volatiles profile of different flower parts and honeys by applying the SPME-GC/MS method, in order to avoid sample alterations from the use of solvents and/or sample heating.

## 2. Materials and Methods

### 2.1. Honey Samples

We chose five samples of* Robinia* honeys produced in 2011 according to two criteria: the geographical origin and the high score obtained for sensory parameters. We used samples coming from five sites representative of the main Italian production areas. A panel of experienced personnel, trained specifically for honey sensory tests, verified the conformity of each sample with the unifloral* Robinia* honey profiles. Moreover, we performed both qualitative and quantitative melissopalynological analysis on each sample according to Louveaux et al. [13] and results confirmed their belonging to the declared category.

### 2.2. Flower Samples

**Figure 1:**
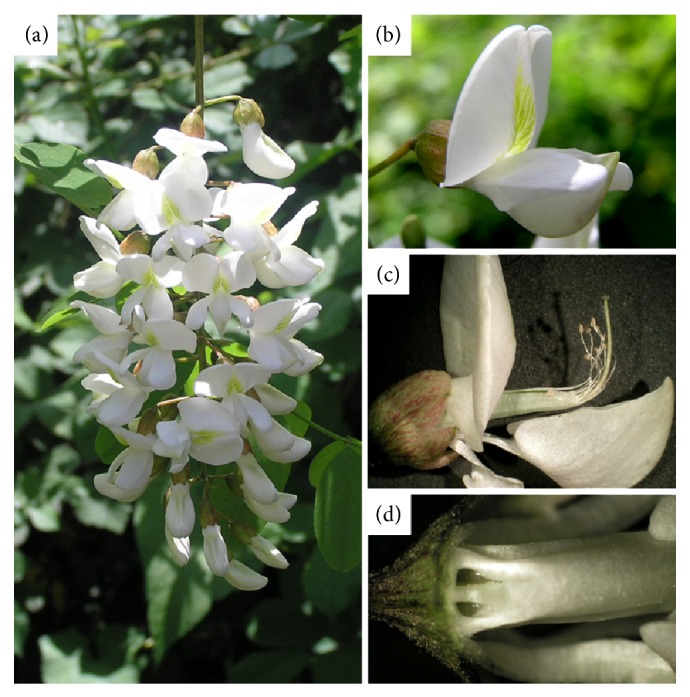
*Robinia pseudoacacia* inflorescence (a) and details of flowers: papilionaceous corolla (b), staminal column of ten stamens and a single pistil from an opened carina (c), and nectar at the base of the corolla (d).

Flowers of* R. pseudoacacia* L. (Fabaceae) consist of a white or light pink papilionaceous corolla with 5 petals: a standard petal (vexillum), two wing petals (alae), and two keel petals (carina) (Figures 1(a) and 1(b)). The carina encloses the staminal column of ten stamens and a single pistil (Figure 1(c)). Nectar is generally secreted in the nectary at the base of the corolla (Figure 1(d)). During the flowering peak we collected open flowers on Monte Somma-Vesuvio (Naples, Southern Italy) and immediately brought them to the laboratory. Different organs of 10 flowers were separated and put in three distinct 15 mL vials: a first vial with stamens and pistils, a second one with petals, and a third one with the remaining joined parts: receptacles, calyxes, and nectaries. Nectar was collected on 200 flowers using glass capillaries, transferred to a 15 mL vial and diluted with 100 *μ*L of water (Milli-Q water purification system, Millipore).

### 2.3. Volatile Extraction and Analysis

For flower volatile extraction we followed Flamini et al. [10] using a fiber DVB-CAR-PDMS 50/30 *μ*m (Supelco, Belfonte, USA). Samples were kept at a 25°C equilibrium temperature for 20 minutes of equilibrium time on a stirrer. After equilibrium, the fiber was exposed to the headspace for 15 minutes and then transferred to the injection port of the GC/MS system for 10 minutes at 230°C. For GC analyses we used a gas chromatograph QP5050 (Shimadzu, Milan, Italy), with a Supelcowax TM10 capillary column (Supelco, Belfonte, USA; 60 m × 0.32 mm, 0.5 *μ*m). Applied conditions consisted of Helium carrier gas at 1.4 mL/min, with an initial pressure of 52 kPa. Column temperature was held at 40° for 4 min, raised to 240°C at 3.5°C/min, and then kept for 3 min. For MS analysis, an electron ionization system was used with ionization energy of 70 eV, electron mass spectra recorded in the 30–250 mass range, with a scanning speed of 0.4 scans/s.

For honey volatile extraction, we followed Soria et al. [14] by using a fiber DVB-CAR-PDMS 50/30 *μ*m (Supelco, Belfonte, USA). We prepared five 10 mL vials with 2000 mg (±0,001) of each honey sample, mixed with 1 mL of water (Milli-Q). Samples were kept at an equilibrium temperature of 60°C for 15 minutes on a stirrer. After equilibrium, the fiber was exposed to the headspace for 30 minutes and transferred to the injection port of the GC/MS system for 10 minutes at 230°C. For GC/MS analyses, conditions were the same as reported above. Compound identification was made by comparison of RT and MS spectra with pure reference compounds and the spectra reported in the NIST 147 Library. A matching higher than 90% and the presence of diagnostic fragments in the spectra were used to identify compounds.

## 3. Results and Discussion

SPME-GC/MS is a method already applied to investigations of flower fragrances [10, 15, 16] as well as honey aromas [6]. The innovative approach of this work consisted of analyzing different flower parts and honey from a single botanical source at the same time, to detect those flower parts contributing to the compounds forming the characteristic blend of the corresponding honey. Moreover, this approach provides a reliable way to choose among volatiles markers, originally related to the plant, to better assess honey botanical origin and authenticity.

### 3.1. Honey

**Figure 2:**
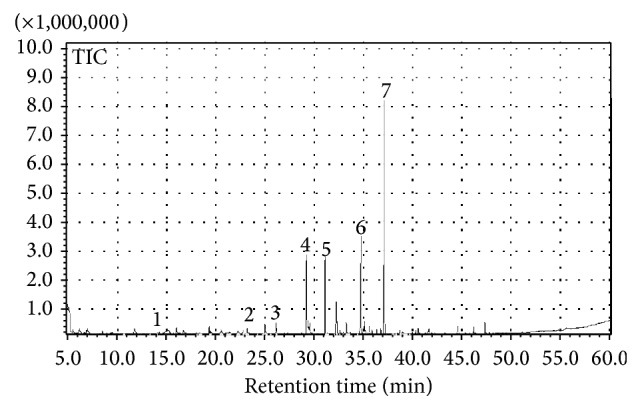
SPME-GC/MS traces of volatile fraction of black locust honey. (1)* 3-Buten-1-ol-2-methyl*; (2)* 3-buten-1-ol-3-methyl*; (3)* 2-buten-1-ol-3-methyl*; (4)* nonanal*; (5)* cis-linalool-oxide*; (6)* linalool*; (7)* hotrienol*.

The aroma profile of* Robinia* honey (Figure 2) shows a characteristic blend, where terpenes are dominant. Other detected compounds were derived from fatty acids (alcohols, aldehydes, and ketones). We identified a total of 70 compounds by SPME-GC/MS analysis from 5 samples of* Robinia* honey (Table 1). A qualitative data evaluation displays volatile compounds of different chemical classes: saturated and unsaturated branched alcohols, aldehydes, ketones, terpenes, and glucose derivatives. The same volatile compounds are mostly recurrent in the five honey samples, independently from their geographical origin, confirming that* Robinia* honey has a distinctive blend of volatile compounds. The analysis of honey profile showed that honey volatiles are mainly represented by high concentrations of terpenes, including linalool,* cis*-linalool oxide and hotrienol. Moreover, there are some branched unsaturated alcohols: 3-buten-2-ol-2-methyl, 3-buten-1-ol-3-methyl, and 2-buten-1-ol-3-methyl, as well as some aromatic alcohols such as benzyl alcohol and 2-phenylethyl alcohol and unsaturated alcohols such as hexanol, nonanol, 3-pentanol-3-methyl, and 1-hexanol-2-ethyl. In the profile emerged also the aldehydes hexanal, octanal, nonanal, decanal, and the branched ketone 5-hepten-2-one-6- methyl.

These results are consistent with several aspects of the literature. Radovic et al. [7] reported* cis*-linalool oxide and heptanal as possible markers of* Robinia* honey, but they also found acetone, furfural, benzaldehyde, and 6-methyl-5-hepten-2-one. Jerković et al. [17] found* cis*-linalool oxide when isolating volatile compounds by hydrodistillation. Since this compound was no more present when applying ultrasonic solvent extraction, they considered it as a thermal artefact. The use of SPME-GC/MS applied by us as well as by Soria et al. [9] is more reliable because it does not involve sample heating. When using CAR/PDMS fiber, Soria et al. [9] found hotrienol,* cis*-linalool oxidem and linalool, as well as 3-methyl-3-buten-1-ol in* Robinia* honeys; when using PA fiber, they found high contents of dihydro-2(3H)-furanone and hotrienol, in addition to minor concentrations of 2-phenylethanol, benzaldehyde, linalool,* cis*-linalool oxide, phenylmethyl ester of acetic acid and several esters.

### 3.2. Flower Parts

**Figure 3:**
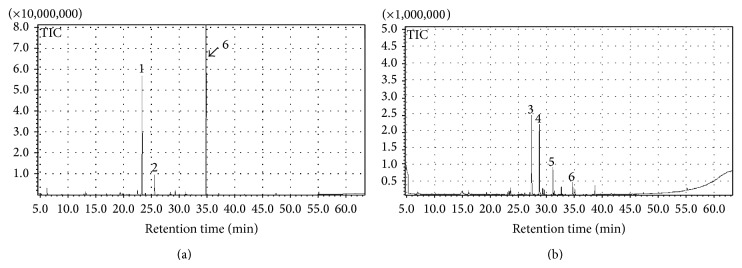
SPME-GC/MS traces of volatile fraction of black locust petals (a) and nectar (b). (1)* Z-Ocimene*; (2)* geranyl nitrile*; (3)* hexanol*; (4)* allo-ocimene*; (5)* cis-linalool-oxide*; (6)* linalool*.

In Table 2 we report the 43 volatile compounds found in different flower parts, namely, stamens, petals, and nectar, and the combined sample of calyxes and nectaries. There is a notable difference between the main compounds characterizing stamens, petals, the combined sample of calyxes and nectaries, and those characterizing nectar alone (Figure 3, Table 2). In fact, the main volatile compounds of black locust floral parts were terpenes, in contrast with the high content of fatty acid derivatives found in nectar.

Stamens were mainly characterized by the terpenes *α*-pinene, D-limonene, linalool, and geranyl nitrile. Fatty acid derivatives were represented by 2-butanone, hexanal, and 1-hexanol. Petals showed high concentrations of monoterpenes as (Z)-ocimene and linalool, followed by geranyl nitrile and *β*-myrcene. Terpenes are again the main compounds found in calyxes + nectaries: *α*-pinene, *β*-pinene, *β*-myrcene, Z-ocimene, geranyl nitrile, and linalool. Low concentrations of 2-butanone, 3-pentanone, and hexanal represented the chemical class of fatty acid derivatives.

As mentioned above, nectar exhibited a high amount of fatty acid derivatives, especially aliphatic alcohols: 1-penten 3-ol, 1-pentanol, (E)-2-penten-1-ol, hexanol, (E)-3-hexen-1-ol, 1-octanol, 1-octen-3-ol, 1-nonanol, and 2-phenylethyl alcohol. Other elements identified in the nectar were aldehydes and ketones: hexanal, 3-octanone, and 5-hepten-2-one-6-methyl. Terpenes as linalool, 1,3,8-p-menthatriene, (4E-6Z)-allo-ocimene, and (4Z-6E)-allo-ocimene complete the sensory profile of nectar.

Xie et al. [18] analyzed whole flowers of* R. pseudoacacia* with similar SPME-GC/MS techniques. They revealed compounds as *α*-pinene, *β*-pinene, and linalool that we found in flower parts. In another study focusing on* Robinia* honey, Truchado et al. [8] analyzed nectar of* R. pseudoacacia* by high-performance liquid chromatography-tandem mass spectrometry. They found a complex flavonoid profile and suggested using flavonoid rhamnosides as floral markers.

SPME-GC/MS is confirmed to be a very reliable technique for honey aroma investigations, because identified volatiles in our results correspond to those obtained in previous analyses. It is also suitable for flower scent analyses, allowing for simultaneous investigations aimed at identifying botanical origins of honey. Nonetheless, caution has to be paid when deciding which floral part to consider (Figure 3) for determining the botanical origin of unifloral honeys. Notwithstanding the fact that bees collect nectar on the flowers, the search of volatile compounds for botanical origin identification should not be confined to nectar but expanded to other floral parts. In fact, in the honey profile we can detect compounds coming directly from the flower but not present in the nectar (hotrienol and *β*-pinene). Such compounds could turn out to be of special interest when selecting floral markers.

## 4. Conclusions

Honey aroma depends on several factors among which the characteristics of nectar are recognized as dominant. In this study, we demonstrated that volatile compounds from other floral parts are transferred to honey. We confirm that SPME-GC/MS technique can be confidently applied for honey aroma investigations. Moreover, we conclude that in the selection of chemical markers to identify the botanical origin of unifloral honeys, in addition to nectar, other floral parts need to be considered.

## Figures and Tables

**Table 1:** SPME-GC/MS analyses of five samples of black locust honeys. Samples have different geographical origins along the Italian Peninsula, but their botanical origin was assessed by melissopalinological and sensory analyses before proceeding with volatile extraction.

Peaks	Retention time	Name	Sample 1	Sample 2	Sample 3	Sample 4	Sample 5
1	5.570	Dimethyl sulfide		0.13		0.43	
2	6.117	Octane	1.12	2.65	0.57	0.62	1.51
3	6.762	Acetone		0.19		0.14	0.36
4	8.541	Nonane		0.48		0.61	
5	9.364	2-Methylbutanal		0.13			
6	9.479	3-Methylbutanal	0.24	0.18		0.31	
7	11.777	2-Pentanone, 3-pentanone		0.28	0.44	1.28	
8	13.952	Pentanal 3-methyl		0.36			
9	14.061	3-Buten-2-ol-2-methyl	0.15	0.17	0.14	0.24	0.23
10	16.002	Hexanal	0.73	0.60	2.53	0.99	0.78
11	16.706	2-Butenal-2-methyl			0.40	0.68	3.18
12	18.033	Butanenitrile-3-methyl	0.60	0.74		0.21	
13	19.325	*β*-Pinene				0.99	
14	20.380	2-Heptanone	0.12				
15	20.521	Hexanal-3-methyl			5.90		
16	20.538	Hexanal-5-methyl		0.40			
17	21.088	Limonene		0.68		0.23	0.89
18	21.440	2-Butenal-3-methyl	0.75		0.70		2.04
19	21.457	Furan-2,3-dihydro-4-methyl		0.54			
20	22.226	(E)-2-Hexenal				0.74	
21	22.765	4,4-Dimethyl-3-oxopentanenitrile	2.11			0.59	
22	22.771	1-Pentene-4,4-dimethyl		3.19			
23	23.160	3-Buten-1-ol-3-methyl	0.55	0.71	0.36	0.87	2.11
24	23.346	uk: 73, 147, 74, 45	0.84	0.86	1.13		0.73
25	24.700	(+)-4-Carene		0.09			
26	24.976	Octanal	2.13	1.69	6.98	2.07	2.08
27	25.875	2-Heptanol	0.11				
28	26.110	2-Buten-1-ol-3-methyl	0.69	0.48	0.50	1.69	2.68
29	26.968	5-Hepten-2-one-6-methyl	0.76	0.42	0.37	0.21	0.24
30	27.307	Hexanol	0.13	0.12	0.22	0.26	1.19
31	28.681	(Z)-3-Hexen-1-ol	0.22				
32	29.214	Nonanal	16.17	13.88	24.11	14.83	16.75
33	29.480	uk: 73, 147, 57, 221	3.13	2.85	2.97		4.56
34	30.426	Dihydro-alpha-terpinyl acetate	0.12				
35	30.806	(E)-2-Ottenale			0.17		
36	31.091	*cis*-Linalool oxide (furan)	9.20	9.47	8.86	9.79	8.89
37	31.298	1-Heptanol			0.98		
38	31.721	7-Octen-2-ol-2,6-dimethyl		0.08			
39	32.207	Furfural	4.44	4.44	4.34	4.93	4.41
40	32.577	1-Hexanol-2-ethyl	1.12	0.80	0.54	0.50	0.87
41	33.231	Decanale	2.95	3.64	2.34	1.59	4.02
42	33.499	Hexane-1-nitro			0.27		
43	33.608	2-Nonanol	0.53	0.36	0.25		0.25
44	34.707	Linalool	15.60	14.18	15.49	16.85	12.80
45	34.992	Lilac aldehyde B	0.44	0.81	0.40	1.02	0.34
46	35.090	3-Pentanol-3-methyl	1.90	1.01	2.70	1.42	1.54
47	35.535	Lilac aldehyde C	0.42	0.90	0.39	1.06	0.32
48	35.829	Lilac aldehyde D	0.19	0.48	0.22	0.62	0.19
49	36.732	Lilac aldehyde A	0.20	0.59	0.25	0.76	0.21
50	37.050	Hotrienol	17.79	20.29	17.67	26.30	19.65
51	38.259	Menthol	0.13	0.09			
52	38.432	Myrtenal				0.11	
53	38.600	(E)-2-Decenal	0.12	0.06	0.88		
54	38.680	1-Nonanol	0.38	0.77	0.38	0.55	0.36
55	38.873	Phenylacetaldehyde	0.66	2.44			0.63
56	39.907	2(3H)-Furanone, 5-ethenyldihydro-5-methyl-	0.22			0.34	
57	40.278	*α*-Terpineol	0.61	0.53	0.37	0.43	0.27
58	40.517	4-Oxoisophorone	0.55	0.33	0.16	0.72	0.41
59	40.607	2-Dodecanol	0.12	0.19			0.17
60	41.624	uk: 133, 68, 151, 59	0.82		0.92		
61	41.944	Linalool oxide (Z-pyranoid)	0.33	0.69	0.11		
62	42.091	1-Decanol		0.09			
63	44.609	*β*-Damascenone	0.71	0.61	0.50	0.91	0.76
64	45.354	Z-Geranylacetone	0.17	0.23	0.18		0.17
65	46.216	Benzyl alcohol	0.88	0.31	0.63	0.86	0.59
66	47.332	2-Phenylethyl alcohol	2.63	1.48	2.49	1.46	2.61
67	48.114	Benzyl nitrile	0.07				
68	48.452	1-Dodecanol	1.50	0.68	0.13		0.19
69	54.276	1-Tetradecanol	0.21	0.14			
70	54.852	Thymol	3.14	1.23			

**Table 2:** SPME-GC/MS analyses of different parts of *Robinia pseudoacacia* flowers.

Peaks	Retention time	Name	Stamens and pistil	Petals	Nectar	Calix and nectaries
1	6.244	4-Methyl-1,3-pentadiene	1.21	1.54		1.32
2	8.956	2-Butanone	5.91	0.13		4.21
3	10.135	Ethanol				
4	11.710	3-Pentanone	0.67			1.05
5	12.880	Butanoic acid-2-methyl-methyl ester	2.73	0.26		
6	13.220	*α*-Pinene	1.33	0.89		20.73
7	16.033	Hexanal	1.36		1.68	1.07
8	16.236	1-Penten-3-ol	0.50		0.89	
9	17.648	3-Butenoic acid-3-methyl-methyl ester		0.06		
10	19.324	*β*-Pinene		0.35		8.56
11	19.429	*β*-Myrcene		1.11		9.42
12	19.876	2-Butenoic acid-3-methyl-methyl ester		0.42		
13	20.827	Methyl tiglate		0.17		
14	21.082	D-Limonene	7.52	0.22		
15	22.516	E-Ocimene	0.79	1.20		
16	23.089	1-Pentanol			1.53	
17	23.271	Z-Ocimene	29.67	30.36		8.34
18	23.496	3-Octanone			3.77	
19	24.230	(3E)-4,7-Dimethyl-3,6-octadienenitrile		0.09		
20	25.311	Cyclopentene, 3-isopropenyl-5,5-dimethyl		0.37		
21	25.540	Geranyl nitrile	17.16	4.25		14.71
22	26.078	(E)-2-Penten-1-ol			0.68	
23	26.144	(Z)-3-Hexen-1-ol, acetate				0.75
24	26.988	5-Hepten-2-one-6-methyl			1.00	
25	27.317	1-Hexanol	1.13		30.92	0.49
26	27.804	(E)-3-Hexen-1-ol			0.14	
27	28.362	(4E-6Z)-allo-Ocimene	0.79	0.52	28.09	0.39
28	28.374	Benzene, methoxy			0.55	
29	28.508	Butanoic acid-3-hydroxy-3-methyl-methyl ester		0.13		
30	29.292	(4Z-6E)-allo-Ocimene		0.99	3.07	3.85
31	29.295	1,3-Cyclohexadiene-1,3,5,5-tetramethyl	0.71		2.29	
32	31.080	1-Octen-3-ol		0.56	10.14	0.58
33	31.110	(E,E)-Cosmene			1.28	
34	31.370	1,3,8-p-Menthatriene		0.17	2.60	
35	32.212	*trans*-Linalool oxide (furan)		0.07	0.36	
36	33.147	*α*-Copaene				1.43
37	34.745	Linalool	28.36	54.86	4.63	23.08
38	35.105	1-Octanol			2.07	
39	37.062	Hotrienol		0.28		
40	37.242	(E)-2-Octen-1-ol			0.16	
41	38.693	1-Nonanol			3.31	
42	47.343	2-Phenylethyl alcohol		0.30	0.57	
43	55.082	Formamide-N-phenyl		0.64		

## References

[1] Kaškoniene V., Venskutonis P. R. (2010). Floral markers in honey of various botanical and geographical origins: a review. *Comprehensive Reviews in Food Science and Food Safety*.

[2] Aronne G., de Micco V. (2010). Traditional melissopalynology integrated by multivariate analysis and sampling methods to improve botanical and geographical characterisation of honeys. *Plant Biosystems*.

[3] Bogdanov S., Ruoff K., Oddo L. P. (2004). Physico-chemical methods for the characterisation of unifloral honeys: a review. *Apidologie*.

[4] Piana M. L., Oddo L. P., Bentabol A., Bruneau E., Bogdanov S., Declerck C. G. (2004). Sensory analysis applied to honey: state of the art. *Apidologie*.

[5] Anklam E. (1998). A review of the analytical methods to determine the geographical and botanical origin of honey. *Food Chemistry*.

[6] Cuevas-Glory L. F., Pino J. A., Santiago L. S., Sauri-Duch E. (2007). A review of volatile analytical methods for determining the botanical origin of honey. *Food Chemistry*.

[7] Radovic B. S., Careri M., Mangia A., Musci M., Gerboles M., Anklam E. (2001). Contribution of dynamic headspace GC-MS analysis of aroma compounds to authenticity testing of honey. *Food Chemistry*.

[8] Truchado P., Ferreres F., Bortolotti L., Sabatini A. G., Tomás-Barberán F. A. (2008). Nectar flavonol rhamnosides are floral markers of acacia (*Robinia pseudacacia*) honey. *Journal of Agricultural and Food Chemistry*.

[9] Soria A. C., Sanz J., Martínez-Castro I. (2009). SPME followed by GC-MS: a powerful technique for qualitative analysis of honey volatiles. *European Food Research and Technology*.

[10] Flamini G., Cioni P. L., Morelli I. (2003). Use of solid-phase micro-extraction as a sampling technique in the determination of volatiles emitted by flowers, isolated flower parts and pollen. *Journal of Chromatography A*.

[11] Alissandrakis E., Tarantilis P. A., Pappas C., Harizanis P. C., Polissiou M. (2011). Investigation of organic extractives from unifloral chestnut (*Castanea sativa* L.) and eucalyptus (*Eucalyptus globulus* Labill.) honeys and flowers to identification of botanical marker compounds. *Food Science and Technology*.

[12] Giovanetti M., Aronne G. (2013). Honey bee handling behaviour on the papilionate flower of *Robinia pseudoacacia* L. *Arthropod-Plant Interactions*.

[13] Louveaux J., Maurizio A., Vorwohl G. (1978). Methods of melissopalynology. *Bee World*.

[14] Soria A. C., Martínez-Castro I., Sanz J. (2003). Analysis of volatile composition of honey by solid phase microextraction and gas chromatography-mass spectrometry. *Journal of Separation Science*.

[15] Barták P., Bednář P., Čáp L., Ondráková L., Stránský Z. (2003). SPME—a valuable tool for investigation of flower scent. *Journal of Separation Science*.

[16] Deng C., Song G., Hu Y. (2004). Application of HS-SPME and GC-MS to characterization of volatile compounds emitted from *Osmanthus* flowers. *Annali di Chimica*.

[17] Jerković I., Mastelić J., Marijanović Z., Klein Ž., Jelić M. (2007). Comparison of hydrodistillation and ultrasonic solvent extraction for the isolation of volatile compounds from two unifloral honeys of *Robinia pseudoacacia* L. and *Castanea sativa* L. *Ultrasonics Sonochemistry*.

[18] Xie J., Sun B., Yu M. (2006). Constituents of top fragrance from fresh flowers of *Robinia Pseudoacacia* L. occuring in China. *Flavour and Fragrance Journal*.

